# Effectiveness of two interventions to optimize expectations in psychosomatic rehabilitation of post-COVID patients: study protocol for an integrative approach

**DOI:** 10.3389/fpsyt.2026.1686494

**Published:** 2026-02-02

**Authors:** Lisa Wedekind, Klaus Hönig, Stephan Frisch, Harald Gündel, Britta Menne, Gottfried Müller, Lena Tepohl, Silke Jankowiak, Rainer Kaluscha, Katja Weimer

**Affiliations:** 1 Department of Psychosomatic Medicine and Psychotherapy, Ulm University Medical Center, Ulm, Germany; 2 Division Medical Psychology, Department of Psychosomatic Medicine and Psychotherapy, Ulm University Medical Center, Ulm, Germany; 3 Rehabilitation Clinic for Psychosomatic, Psychotherapeutic and Internal Medicine Glotterbad, Glottertal, Germany; 4 Rehabilitation Clinic for Psychosomatics, Schlossklinik Bad Buchau, Bad Buchau, Germany; 5 Institute for Research in Rehabilitation Medicine at Ulm University, Bad Buchau, Germany

**Keywords:** biopsychosocial model, expectation, placebo effect, post-COVID syndrome, protocol, psychosomatic rehabilitation, psychotherapy

## Abstract

**Background:**

Most prevalent symptoms of post-COVID syndrome (PCS) are fatigue, shortness of breath, cognitive impairment, and pain. Comorbidities such as depression and anxiety are common. The diagnosis PCS is confirmed if symptoms persist for longer than 3 months and are not better explained by another medical condition. About half of the patients are still not fit for work after psychosomatic rehabilitation. From psychotherapeutic research, it is known that a relationship-based approach is decisive for treatment efficacy. Here, the patient’s expectation that the treatment will help is one central pathway that contributes to symptom reduction and an increase in quality of life. Using positive expectations, respectively, the placebo effect in medical settings has already been proven to be beneficial.

**Objective:**

Evaluation of two expectation-focused interventions for patients with PCS to optimize the rehabilitation process. The interventions aim to focus on positive expectations and imaginations. Thus, the project should lead to further improvement in the rehabilitation of patients with PCS.

**Methods:**

The study implements a 3-arm, parallel group, longitudinal non-randomized controlled, sequential cohort design. The trial is designed to estimate the effect of *1) a 3-session psychotherapeutic expectation-focused integrative, manualized intervention, and 2) a brief intervention where patients are asked to write down their expectations on their own in a journal, compared to 3) a treatment-as-usual-only control condition (usual treatment in rehabilitation, no study intervention)*. Since the predominant symptoms in PCS patients vary a lot, the Work Ability Index (WAI) is considered as a proxy for earning capacity as primary outcomes.

**Discussion:**

The analysis will provide insights into the extent to which the interventions improve PCS. This approach was chosen to enable a larger number of practitioners to provide more specific therapeutic support for patients with PCS. The study serves as proof of principle for further research and development of more effective therapies. It emphasizes the urgent need for interdisciplinary, integrative treatment and research to better understand and manage PCS.

**Clinical trial registration:**

German Clinical Trials Register https://drks.de/search/en, DRKS00034671.

## 1 Introduction

According to the World Health Organization (WHO), the post-COVID syndrome refers to symptoms that persist or newly appear more than three months after a SARS-CoV-2 infection and for which there is no (better) alternative explanation (1).The post-COVID syndrome (PCS) does not manifest itself as a uniform pattern, but consists of more than 200 different symptoms. Studies show that the most prevalent symptoms are fatigue, shortness of breath, cognitive impairment and psychosomatic complaints such as depression, anxiety, pain and post-traumatic stress disorder (2–4). Meta-analyses also carve out symptoms in 30% of patients even two years after COVID-19 (5). Furthermore, in approximately 50% of patients, the ability to work is not restored to its pre-infection level after psychosomatic rehabilitation, although they still seem to benefit significantly from the interventions (6).

Organic causes for PCS symptoms are found in less than 10% of people who seek PCS consultation (7, 8). However, dysfunctional behavioral patterns such as avoidance, endurance, misattribution, irregular breathing, and deconditioning seem to play a crucial role in the development and maintenance of symptoms. This makes the disease particularly interesting from a psychosomatic perspective. Additionally, there are also indications that dysfunctional symptom expectations contribute to symptom persistence (9–11).

An integrative causal model incorporating biological, social, experiential, and psychological factors for the development of PCS has been proposed (12). Expectations play a relevant role in all factors of this model. A recent systematic review with meta-analysis (13) also reports a significantly increased risk of developing symptoms that occur after a SARS-CoV-2 infection due to pre-existing depression or anxiety disorders, underscoring the influence of the psyche in PCS.

Furthermore, some authors suggest that PCS should be considered as persistent somatic symptoms (e.g. 14). This is a collective term for symptoms that cannot be adequately explained by structural diseases or tissue damage and often defy clear classification within conventional dualistic epistemology. The lack of a sufficiently explanatory somatic pathology increases uncertainty, fear, and stigmatization in patients and contributes to fragmented, inappropriate treatment. The vulnerability-stress model is helpful for the understanding of persistent physical symptoms, in which there is an individual, complex biopsychosocial interplay of predisposing, triggering, maintaining and reinforcing factors.

The models discussed above, and the psychological comorbidities indicate the need for psychosomatic-psychotherapeutic, multimodal, interdisciplinary treatment for patients with PCS. Accordingly, the AWMF S1 guideline for the treatment of PCS in Germany points out that psychotherapy should be offered to optimize coping strategies, treat psychological comorbidity, manage pain and teach relaxation techniques, for example. AWMF stands for “Arbeitsgemeinschaft der Wissenschaftlichen Medizinischen Fachgesellschaften”, which translates to Association of Scientific Medical Societies in Germany. The AWMF S1 guideline is a clinical guideline based on expert consensus, typically used when scientific evidence is insufficient or inconclusive. It offers guidance based on expert opinions to help healthcare professionals make informed decisions in the absence of stronger evidence (15).

A recent systematic review (16) of psychotherapy in patients with PCS found positive effects on depression, anxiety, quality of life, fatigue, and pain during treatment in most studies – with ten out of twelve studies investigating multimodal therapies including psychotherapy. The only randomized controlled trial (17) reported a significant improvement (only) in fatigue with a high effect size as a result of four months of cognitive-behavioral therapy. A considerable number of psychotherapy studies in PCS are currently being conducted or prepared (e.g., 18, 19). In addition, studies in the field of rehabilitation medicine should be mentioned in this context, which confirm the positive effects of multimodal psychosomatic therapy (20, 21). Here, PCS patients seem to benefit significantly from rehabilitative, psychotherapeutic measures – including exercise therapy aimed at improving body awareness.

But what makes psychotherapy effective? In contrast to the medical metamodel, the contextual metamodel (22) describes a relationship-based, cross-methodological approach as crucial for treatment success. The three key factors of this model are the patient’s expectations, a genuine patient-therapist relationship, and specific psychotherapeutic treatment techniques. The patient’s expectation that the treatment will help is, therefore, one pathway that contributes to symptom reduction and an improvement of quality of life. The social context of treatment should be characterized by trust, understanding, and competence. Both the therapist and the patient contribute with their personal assumptions (for a deeper understanding, see 23). In addition to the therapeutic mechanism, the social connection, and the effects of the psychotherapeutic guideline procedure, creating positive expectations about the current situation and treatment is crucial.

Using positive expectations in medical settings has repeatedly been shown to be helpful (24, 25). For example, Rief et al. (26) and Auer et al. (27) have shown that “psychological preparation” using positive expectations influences health outcomes after cardiac bypass surgery: patients who have received an intervention to optimize expectations, reported an improved recovery six months after surgery with a decrease in health impairments, cardiac anxiety, pro-inflammatory cytokine concentrations, lower hormonal stress reactions, a shorter stay in hospital and an improvement of quality of life, as well as “fitness for work” (hours per working day). It is therefore clear that patients´ expectations of treatment have a significant influence on the success of interventions, regardless of the type of disease (28–30), and expectations can significantly alter the course of treatment (31). Studies even show that negative expectations can have a dysfunctional effect on the body and psyche or even promote PCS (32–34). Interestingly, Funk et al. (35) show that patients with persistent symptoms after COVID-19 gave positive feedback on an expectation management intervention in a qualitative study. This effect should also be used in our planned quantitative study.

In summary, the persistent symptoms of PCS appear to be maintained primarily by psychological mechanisms such as patient expectations. Those could be changed through targeted interventions, which in turn lead to symptom improvement. Thus, the following research questions will be examined and hypotheses were formulated:

I. Does the addition of a psychotherapeutically guided intervention aimed at optimizing realistic and optimistic expectations regarding rehabilitation result in a significantly greater improvement in Work Ability Index (WAI) scores from pre- to post-treatment compared to standard treatment alone in patients with PCS undergoing rehabilitation?

Hypothesis: It is hypothesized that patients who receive the psychotherapeutically guided intervention in addition to psychosomatic rehabilitation will show a significantly greater improvement in WAI scores from pre- to post-treatment compared to patients who receive psychosomatic rehabilitation only.

II. Does an additional simplified version of the intervention, based on the same manual as the psychotherapeutically guided intervention and in which patients work independently on their expectations, result in a significantly greater improvement in WAI scores from pre- to post-treatment compared to standard treatment alone in patients with PCS undergoing rehabilitation?

Hypothesis: It is hypothesized that the simplified and less resource-intensive intervention in addition to psychosomatic rehabilitation will show a significantly greater improvement in WAI scores from pre- to post-treatment compared to patients who receive psychosomatic rehabilitation only.

## 2 Materials and methods

The protocol is based upon the SPIRIT reporting guidelines (36) and follows Allwang et al. (18) and Nickel et al. (37).

### 2.1 Objectives

The aim of the study is to investigate the effectiveness of two psychotherapeutic interventions that focus on positive expectations of patients with PCS during a psychosomatic rehabilitation program compared to treatment as usual alone (usual treatment in rehabilitation, no study intervention). Psychosomatic rehabilitation for patients with PCS in Germany follows a structured, multidisciplinary approach tailored to address both the persistent physical and psychological effects of the condition. Psychosomatic rehabilitation measures are paid for by the pension insurance provider, which checks compliance with the structured treatment. Rehabilitation begins with a thorough initial examination, which includes medical examinations, psychological screening, and functional assessments to identify the full range of symptoms, such as fatigue, shortness of breath, cognitive impairment, anxiety, and depression. Based on these assessments, structured treatment is individually tailored and comprises various therapeutic components designed to promote both physical and psychological recovery. The rehabilitation program typically combines physical therapy to restore endurance and muscle strength, especially in cases of post-viral fatigue and respiratory difficulties. Psychotherapy is frequently used to treat mental health issues such as anxiety, depression, and PTSD, which are common in patients with PCS. Relaxation techniques, such as progressive muscle relaxation or mindfulness meditation, are used to reduce stress and improve overall emotional regulation. Additionally, social and vocational counseling is integrated to support reintegration into working life and everyday routines and to help patients overcome challenges related to returning to work or dealing with social interactions. The rehabilitation program usually lasts between 3 to 4 weeks with intensive daily sessions, but may be extended depending on the patient’s individual progress and needs. Throughout the process, patients are closely monitored and the rehabilitation plan is adjusted as necessary. After completing treatment, patients often continue to receive care, for example through outpatient therapies or regular check-ups, to ensure lasting recovery and treat any remaining symptoms. The holistic approach aims to improve both physical functioning and mental health, ultimately enhancing the patient’s quality of life and ability to return their normal activities.

### 2.2 Study setting and responsibilities

The study was designed and is being coordinated by the Department of Psychosomatic Medicine and Psychotherapy, Ulm University Medical Center, Ulm, Germany. The institution is being advised and supported in data collection, data analysis, and reporting by the Institute for Research in Rehabilitation Medicine at Ulm University, Bad Buchau, Germany (“IfR Ulm”). The study is being conducted at the rehabilitation clinics Glotterbad, Glottertal, and Schlossklinik, Bad Buchau, Germany. All participating study therapists are psychotherapists or physicians (in training or licensed) and undergo structured training to perform the interventions according to the manual developed by the authors of the study. The training takes place in person at both study locations. The session lasts approximately 3 hours in total, including a 30-minute break. The training is conducted by a psychologist experienced in the field of placebo research (KW) and a licensed psychotherapist (LW). First, the study therapists are informed about the scientific background. Then, the study is presented and the therapist manual is reviewed together. Individual exercises are practiced in pairs. At the end of the training, the study therapists receive a detailed overview of the study design and next steps. To ensure that standard therapy and interventions are not influenced by the training of therapists, therapists are trained only before the respective intervention group. However, influence cannot be completely ruled out, as the principles of the interventions are generally known to therapists.

Regular supervision and peer consultation sessions are conducted by the Department of Psychosomatic Medicine and Psychotherapy at Ulm University Medical Center in both rehabilitation clinics.

The study is funded by the German Pension Insurance Fund of Baden-Württemberg, whose primary interest lies in maintaining and restoring patients’ ability to work. However, it has no influence on the study design, study implementation, or data analysis.

### 2.3 Design

The multicenter, longitudinal, quantitative trial employs a quasi-experimental, non-randomized sequential cohort design with three arms in a real-life design. The entire project will run from January 01, 2024, until expected June 30, 2027.

### 2.4 Participants

Patients with post-COVID syndrome (PCS) who are being treated at participating rehabilitation centers are included (ICD-10 codes U08.09 and U09.9; at least one persistent PCS symptom that persists for at least 12 weeks after the initial infection). Eligible patients are adults of working age, generally between 18 and 65 years old, as rehabilitation measures in Germany are financed by the pension insurance provider in order to maintain and restore patients’ ability to work. They must have sufficient knowledge of German to complete the questionnaires and understand the intervention and must be able to give written informed consent. Study participants receive comprehensive information about the study. Potential benefits and effects as well as possible side effects are explained. Additionally, participants are informed about data security and protection, and their rights as study participants. Study staff obtains written informed consent prior conducting baseline assessments. Patients with severe psychiatric conditions according to ICD-10 (e.g., psychotic symptoms, dementia/Alzheimer’s disease, known substance dependence and abuse [except nicotine]) or current suicidal ideation will be excluded. The following demographic variables are collected as part of the study: age, sex, immigration status, family/relationship status, children, place of residence (village, small town, medium-sized town, large city), level of education, occupational group, and employment.

### 2.5 Interventions

#### 2.5.1 Explanation for the choice of comparators

**Figure 1:**
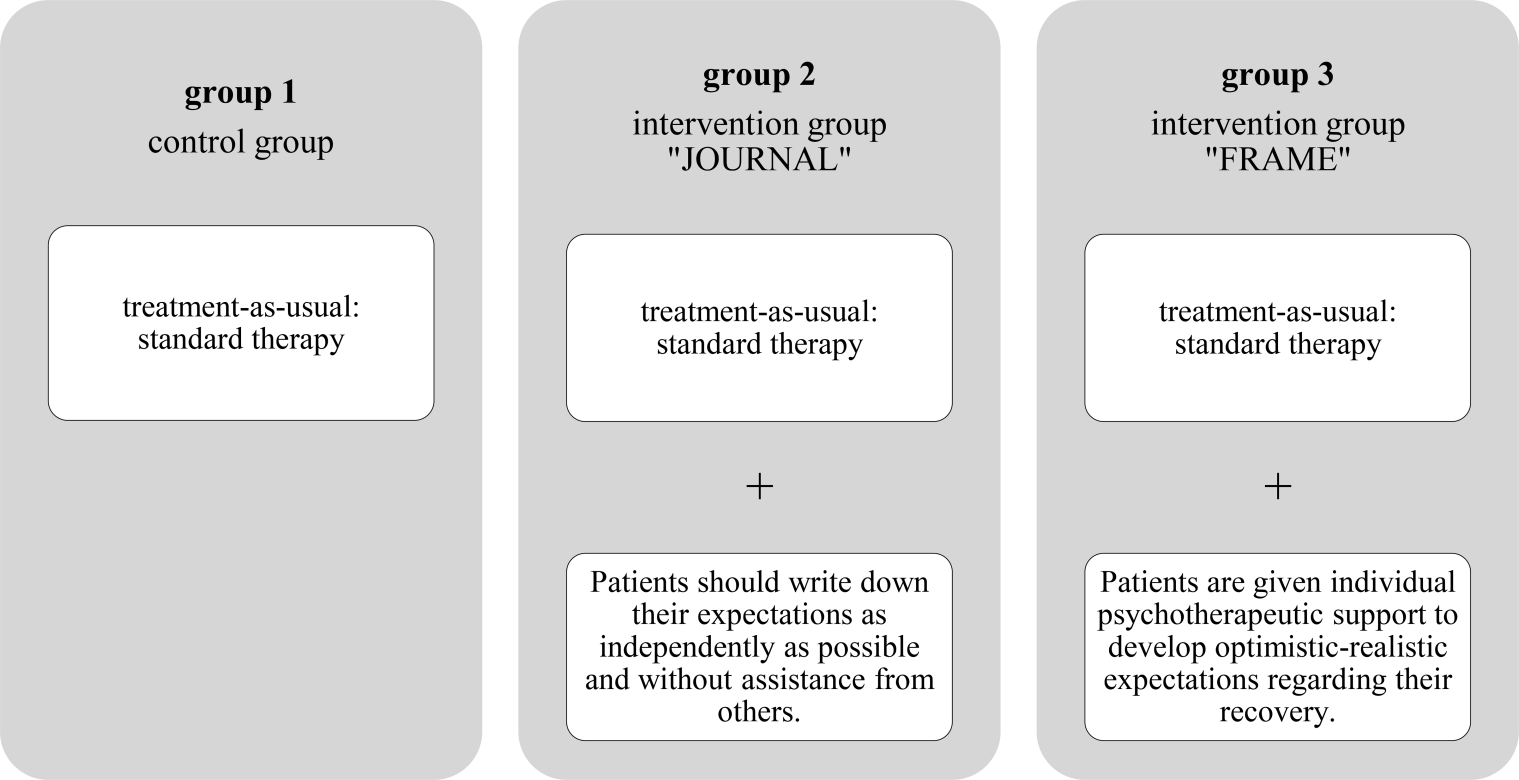
The three groups of the study.

Throughout the study, all three study groups participate in regular psychosomatic rehabilitation and receive treatment as usual (TAU) in accordance with the guidelines of the German pension insurance for patients with PCS. The control group (group 1) receives only TAU, while the other two groups additionally receive one of the two interventions (group 2 = JOURNAL group; group 3 = FRAME group). Although participants are assigned to groups according to a sequential cohort design, systematic differences or changes in symptom burden should be minimal and attributed to group affiliation and whether a study intervention was received. Which group participants are in depends solely on the timing of their rehabilitation, not on other systematic differences. (see **Figure 1**). Patients are informed about the study on the first day in the rehabilitation hospital. All treating providers are aware of the participants’ condition assignments.

#### 2.5.2 Intervention description

During psychosomatic rehabilitation, the following points are discussed with all patients as standard and are also important for participation in the study:

- Openness to psychotherapeutic interventions
- Healthy, active lifestyle, considering the patient’s ability to manage their own strength, reduction of excessive avoidance behavior
- Acceptance of illness

The complete manual on the interventions will be published separately. Therefore, the content of the interventions will only be described briefly below.

##### 2.5.2.1 Group 2 – Intervention group “JOURNAL”

Patients in group 2 receive TAU and an extra intervention consisting of two short appointments (sessions) lasting no more than 20 minutes. The main point of this intervention is that patients should write down their expectations and imaginations as independently as possible and without assistance from others, e.g. a therapist. Patients in the JOURNAL group are given an accompanying booklet as part of the study, which summarizes the most important information from the individual sessions. The booklet also contains illustrations and worksheets and is the same as in the FRAME group. The booklet is distributed to patients in group 2 on the first days of the rehabilitation by a research assistant who was trained by the team in Ulm. Patients are asked to reflect on their individual expectations of therapy in writing. The individual sessions in which the booklet is discussed include specific reflection questions and exercises designed to help patients process their personal expectations and challenges. In detail, the booklet contains two tasks in which patients are asked to reflect on their expectations, an imagination exercise, and a list of enjoyable activities for after rehabilitation. When leaving the rehabilitation clinic, patients are allowed to take the journal home with them. Only a documentation form completed by the research assistant remains at the clinic.

##### 2.5.2.2 Group 3 – Intervention group “FRAME”

Patients in group 3 also receive TAU and a manualized additional intervention consisting of three appointments (see **Table 1**). Patients receive the same booklet as in group 2, but are given therapeutic guidance on how to work through, and develop the content and carry out the imagination exercise together with the therapist. The psychotherapeutic FRAME intervention was developed through an interactive process by a team of interdisciplinary experts (psychologists experienced in inducing placebo effects through expectations, psychotherapists specializing in psychodynamics and cognitive behavioral therapy, hypnotherapists, and medical specialists in psychosomatics, psychiatry, and neurology) and is based on various approaches. The focus of this intervention is primarily on positive expectations (benefit expectations, personal control expectations, side-effect and coping expectations). The structure of the intervention is based on the manual Psy-HEART (38). General therapy principles – such as tangential conversation, avoiding confrontation with resistance and interpretations, selective-authentic responses, integration of psychoeducational elements and working on expanding the individual disease model – were adopted from the psychodynamic manual PISO (39) and are based on the findings of intersubjective systems theory, relational psychoanalysis and attachment theory. Furthermore, hypnotherapeutic elements are integrated, such as an imagination exercise (40). The core element of the intervention is affective-body-oriented work with inner images (imagination exercise).

**Table 1:** Session focus, interventions and duration for each therapeutic session in the FRAME condition.

Session	Session focus and interventions	Duration
1	− Therapeutic relationship − Explanation of the procedure and aim of the intervention, handing out the accompanying booklet − Expectation-focused anamnesis: The current situation and current complaints are discussed briefly. The focus should be *on identifying expectations*. − Focus on benefit expectations: The biopsychosocial disease model should be introduced or taken up (if the patient already knows this) in order to build up the benefit expectations based on this. Expectations regarding the benefits of rehabilitation can be discussed in relation to the current symptoms: *Information about symptom improvement, increase in quality of life and a positive-realistic recovery process through rehabilitation should be addressed.* − *Imagination exercise* (outlook): Considering personal needs, interests, and wishes, the patient should, by the next session, *imagine a picture expressing success and an improved quality of life six months after rehabilitation*. − End of the session: Summarizing what has been achieved and looking ahead to the next two sessions.	50–60 minutes
2	− Beginning o Questions and comments on the patient information (*accompanying booklet*) should be briefly clarified and discussed. By working with a metaphor, control expectations should be increased. − Focus on control expectations (=“control beliefs”) o With reference to part of the first session (○ Personal control expectations) and using a metaphor, the introduction to the imagination exercise is paved. − Increase control expectations with the help of an imagination exercise o The extent to which inner images or imaginations can be used constructively in sports psychology will be illustrated using an example and the fact that this technique is also used in the intervention will be discussed. o In the following, the imagination exercise will be developed using the “VAKOG scheme” (Peter et al., 2015) in a first step (scene from the past). VAKOG stands for seeing (visual), hearing (auditory), feeling (kinesthetic), smelling (olfactory) and tasting (gustatory). o In a second step, the imagination exercise should be carried out and then debriefed. − Conclusion o Summarize what has been worked out and look forward to the last appointment. o The advantages of rehabilitation should be emphasized again at this point. o The imminent end of the intervention should be marked.	20–30 minutes
3	− Greeting with reflection on expectations: The patient should take up their expectations formulated in session I, read them through and reflect on them with the therapist. − Deepening, repetition, practicing of the imagination − Focus on side effect and coping expectations − Pleasant activities: Suggestions for pleasurable activities should be discussed with the patient using a provided list. The patient should select the examples that they consider suitable for themselves to build positive expectations for the future. − Summary and conclusion of the intervention	20–30 minutes

A therapeutic example from the PoCo-FRAME manual is described below. It is based on Laferton et al. (38, p. 24), has been translated into English, adapted to PCS, and supplemented.

Therapist: “I am very pleased that you have agreed to participate in our study. I believe you’ve made a good decision regarding the course of your rehabilitation. The goal of our collaboration is to explore your expectations regarding the success of your rehabilitation. Research has shown that patients who believe in the effectiveness of a treatment often experience better health outcomes afterwards. I´ll help you benefit as much as possible from this effect. I see my role as a confident companion on your journey. There may be times when you have questions or uncertainties — and we’ll address these together. It’s particularly important that we focus on your personal perspective. Please feel free to share your expectations in detail. Today, we’ll primarily focus on what you expect from the rehabilitation process. So, how about you start by telling me about your current situation and your expectations?”

The intervention is carried out as individual therapy in three sessions with one session per week (see **Table 1**). In addition, the patients in the FRAME-group receive a booklet summarizing the most important information from the individual sessions. The booklet also contains illustrations and worksheets that can be completed during or between sessions. Patients should bring the booklet to each session and read the information during the appointments. Therapists receive a documentation form for each patient, which must be completed. After each session, therapists must document which components of the intervention patients have completed.

#### 2.5.3 Criteria for discontinuing or modifying allocated interventions

In the event of an acute or significant deterioration of symptoms, including the onset of suicidal tendencies, the intervention will be either modified or discontinued. The decision on this matter rests with the treating therapist or physician at the rehabilitation clinic, who will discuss this with the patient. In such cases, the rehabilitation clinic will initiate appropriate therapeutic treatment. Participants also retain the right to withdraw from the intervention at any time and for any reason, without providing justification. Withdrawal from the study – for whatever reason - has no impact on the rehabilitation measures for patients.

Selection bias cannot be ruled out in studies, especially in real-life studies. The interventions are designed in such a way that we expect a low dropout rate and plan to recruit the calculated sample size. Due to data protection regulations, the data of patients who have dropped out cannot be used for *post-hoc* analysis.

#### 2.5.4 Strategies to improve adherence to interventions

As part of the participant information and informed consent process, patients are informed about the importance of consistent and active participation in therapy and intervention sessions. Treatment adherence is assessed using standardized checklists that list the intended content of each session. Therapists document which components of the manual were implemented and which were omitted. In cases where certain elements could not be delivered, the underlying reasons are noted – for example, if the session content was too demanding for the participant due to difficulties with concentration.

#### 2.5.5 Relevant concomitant care permitted or prohibited during the trial

All forms of medical treatment and therapeutic interventions are permitted throughout the study and will be systematically documented. All patients will receive TAU during the psychosomatic rehabilitation program. This treatment as usual can vary between rehabilitants, as the patient’s stress limit and symptoms are always considered when planning therapy.

#### 2.5.6 Provisions for post-trial care

It is assumed that participants will benefit from the intervention, resulting in an improvement in PCS symptoms. Adverse effects related to study participation are not anticipated.

#### 2.5.7 Outcomes

Since the predominant symptoms in patients with PCS vary considerably and the main goal of rehabilitation is to maintain or improve work ability, the Work Ability Index (WAI) is considered the primary endpoint for measuring work ability. The WAI ranges from 7 points (unable to work) to 49 points (full work ability), with higher scores indicating better subjective work ability. Consequently, increases in WAI scores reflect improvements in perceived work ability, whereas lower scores indicate reduced work ability. International studies conducted in recent years in Europe, South America, and Asia show high internal consistency between 0.72 and 0.83. The WAI also correlates highly and significantly with all scales of the Short-Form Health Survey (SF-36) and the General Health Index (r between 0.5 and 0.8) (41). It is therefore a reliable and proven tool in rehabilitation research.

Secondary outcomes include: WHO Disability Assessment Schedule 2.0 (WHO DAS 2.0), PCS symptoms (adapted Regensburg COVID documentation, ReCoRD), somatic symptoms (Patient Health Questionnaire 15, PHQ-15 and Somatic Symptom Disorder B-criteria Scale, SSD-12), depressiveness (Patient Health Questionnaire-9, PHQ-9), anxiety (Generalized Anxiety Disorder Scale, GAD-7), fatigue (Fatigue Scale for Motor and Cognitive Functions, FSMC), post-exertional malaise (DePaul Symptom Questionnaire), expectations regarding rehabilitation and its outcome (Treatment Expectation Questionnaire, TEX-Q), self-efficacy expectations (SWE), and quality of life and general health status (2 items).

In addition, personality questionnaires are used to identify correlations between personality functioning and PCS to provide patients with the best possible psychotherapeutic support in the future. The transdiagnostic personality structure (Operationalisierte Psychodynamische Diagnostik – Strukturfragebogen Kurzform, OPD-SFK) and a scale for perfectionism (Frost Multidimensional Perfectionism Scale – Deutsche Version, FMPS-D) are used for this purpose.

Objective measurements include computer and paper-based tests to assess cognitive performance, such as attention span, memory, and reaction times (Corsi Span Test and Color Stroop Test), 6-minute walk test, blood pressure, and 5-minute pulse measurement at rest to assess heart rate variability. Further measurements include clinical assessments of rehabilitation outcomes and medical evaluation of the ability to work (discharge letter) by the physician. The cognitive assessments are not part of routine clinical evaluations; they are specifically implemented for the purposes of this study.

A comprehensive range of outcomes was selected by a multidisciplinary research team to ensure the future provision of optimal support for patients.

The questionnaires are used in all three groups at the beginning baseline (T1; baseline), at the end of rehabilitation (T2), and six months after rehabilitation program (T3). Rehabilitation usually ends after 3 to 4 weeks. The final measurements for the study are taken during the last psychotherapy session in the final week.

Participants with PCS may experience difficulties using the screen and concentrating. To ensure their comfort and reduce any burden, we have made several adjustments available. Participants have the option to take breaks as needed, complete the questionnaires at their own pace, and even continue at a later time if necessary. Additionally, paper versions of the questionnaires can be provided upon request, and participants can also complete them in their rooms if this is necessary. The assessment sessions are expected to take between 70 and 90 minutes per assessment, depending on the individual condition and needs of the patient.

#### 2.5.8 Participant timeline

A schematic schedule for participants is depicted in **Table 2**.

**Table 2:** Schedule for participants (T1, T2, T3 = time 1, time 2, time 3).

Assessments	T1 on admission to rehabilitation	T2 on discharge from rehabilitation	T3 6 months after rehabilitation
questionnaires	X (online in the clinic)	X (online in the clinic)	X (online from home)
cognitive tests	X (online in the clinic)	X (online in the clinic)	X (online from home)
blood pressure	X	X	
heart rate variability	X	X	
6-minute walk test	X	X	
discharge letter		X	

#### 2.5.9 Sample size

A total sample of N = 84 participants will participate in one of three groups: a control group receiving treatment as usual only (TAU), a JOURNAL group, and a FRAME group, with 28 individuals in each group. Case numbers are based on the following facts: In general, hardly any studies have been published on the effectiveness of psychosomatic rehabilitation measures for patients with PCS (42). Initial studies show medium to large effects, depending on the outcome variable. For example, effects were observed for somatoform complaints (d=0.60) and the 6-min walking test (d=1.22) (6). No study could be found that showed effect sizes for changes in earning capacity or ability to work. Rief et al. (26) found a large effect size (approximately d=1.0, calculated from the data in the article) for improvements in limitations and the subjective assessment of ability to work in hours (approximately d=2.0, calculated from the data in the article) through the intervention to optimize expectations in patients undergoing bypass surgery. As the patients in this study are already undergoing psychosomatic rehabilitation treatment, the intervention is an add-on that may not show a statistically large effect (Cohen’s d > 0.8). Therefore, the sample size should be large enough to detect a medium effect. Furthermore, it should be possible to test the effectiveness of each intervention compared to the treatment as usual (TAU) group. For a 2x2 analysis of variance with repeated measures (ANOVA; 2 groups x 2 time points) for the change in the main outcome measure (e.g., WAI) from admission to discharge, with a mean effect size of f=0.25 and a power of 0.95, N = 54 patients are required for the comparison of TAU with one of the intervention groups (calculated with G*Power 3.1.9.6; freeware from Kiel University). In order to compare the second intervention group with the TAU group, further N = 54/2 = 27 patients are required in this group. In total, this equates to N = 81 patients. To be able to divide these patients between the two clinics and the three groups, a total of N = 84 patients are required, for whom at least admission and discharge data are available. This means that the sample is also sufficiently large for 2x3 ANOVAs (2 groups x 3 time points; N = 36 required). In summary, we plan to conduct two 2×2 ANOVAs, specifically using a mixed-design ANOVA (two-factor ANOVA with one repeated-measures factor and one between-group factor). With an estimated drop-out rate of 20%, 100 patients must be recruited.

#### 2.5.10 Recruitment

Patients are recruited through participating rehabilitation clinics that specialize in treating PCS. Patients receive information about the study either in advance by post or upon admission to the clinic. During the admission interview, a therapist or physician establishes whether the inclusion and exclusion criteria are met, and asks patients if they wish to participate in PoCo-FRAME. The three groups (two interventions and one control) are recruited in clusters, with a two-week washout phase in each of the two rehabilitation clinics. This means that only one group is recruited and examined at a time, until that group is complete. This reduces confounding factors, such as disappointed patients in the control group talking to patients in an intervention group.

### 2.6 Assignment of interventions: sequential cohort design

In order to recruit enough patients within a reasonable timeframe, the study will be conducted at two locations. Due to different treatment concepts at each rehabilitation clinic, this will also increase the study’s external validity. Patients will be recruited into the two intervention groups and the control group in clusters, with a wash-out phase in each clinic. This means that only one group will be recruited and examined at a time, and the next group will only be recruited two weeks after the previous group has been fully discharged. This reduces confounding factors, such as disappointed patients in the control group talking to patients in an intervention group. The random allocation of patients is based on their start date on the rehabilitation program.

### 2.7 Data collection and management

#### 2.7.1 Plans for assessment and collection of outcomes

The variables of interest are assessed via digital self-assessment questionnaires via the online platform Unipark (Tivian XI GmbH, Cologne, Germany).

#### 2.7.2 Plans to promote participant retention and complete follow-up

Participants are free to withdraw from the study at any time, and without providing a reason. In such cases, no further outcome data will be collected. To encourage completion of the six-month follow-up assessment, participants will receive reminder emails one and two weeks after receiving the initial invitation to complete the follow-up questionnaire. If the questionnaire remains incomplete after three weeks, a final reminder will be issued via telephone.

#### 2.7.3 Data management

As data collection is conducted digitally via Unipark, information is entered directly by participants, therapists and research assistants. The data is then exported from the platform’s database in CSV or SPSS format.

#### 2.7.4 Confidentiality

Participant data is collected in pseudonymized form. For statistical evaluation, the data is transferred to the analysis software R and SPSS. Access to the keys that link pseudonyms to individual identities, as well as to the digitally recorded health-related participant data, is restricted exclusively to authorized study personnel at the respective rehabilitation clinics.

### 2.8 Statistical methods

#### 2.8.1 Statistical methods for primary and secondary outcomes

Demographic data will be used to describe study participants and reported as means and standard deviations or median and interquartile range, where appropriate. The change in primary and secondary outcomes as well as objective measures from the beginning to the end of rehabilitation for each intervention group individually with the control group will be analyzed with 2x2 ANOVAs (2 time points x 2 groups). The longterm effects after six months will also be analyzed for each intervention group with separate 3x2 ANOVAs (3 time points x 2 groups). Since this is the first study involving these interventions in patients with PCS, prior correction will not be used, but will be discussed appropriately in the publication of the results. For the exploratory evaluation of personality measures, we plan to calculate predictor analyses, such as regression analyses to predict changes in primary and secondary outcomes.

#### 2.8.2 Methods in analysis to handle protocol non-adherence and any statistical methods to handle missing data

Study adherence is defined as participation in all sessions according to group assignment and completion of at least 80% of the tasks in the booklet. Non-adherence is noted if participants fail to complete more than 20% of the tasks. Therapists use a standardized checklist to document which components were completed or omitted and note the reasons for any deviations (e.g., if the content of the session was too demanding). Adherence is monitored by attendance at sessions, documentation by therapists, and self-reports by patients. Recruitment continues for each group and clinic until the required 14 patients have met the adherence criterion.

The primary outcome, the WAI, must not contain any missing values. All other questionnaires are evaluated in accordance with their manuals, and individual missing values are treated or imputed as described in the manuals.

#### 2.8.3 Plans to give access to the full protocol, participant level-data, and statistical code

The study protocol is published in this article, and final statistical analyses will be reported in the publications of study results. Due to privacy policies, patient data cannot be published, but aggregated data can be made available to researchers with a legitimate interest.

### 2.9 Oversight and monitoring

#### 2.9.1 Composition of the coordinating center and trials steering committee

The study is coordinated by the Department of Psychosomatic Medicine and Psychotherapy in Ulm. Statistical analyses are carried out at this department and at the Institute for Rehabilitation Medicine in Bad Buchau. The psychotherapeutic interventions will take place at the psychosomatic rehabilitation clinics Glotterbad and Schlossklinik Bad Buchau in Germany. At each study site, 42 participants will be included. The coordination team meets regularly once a week. Full staff meetings are held monthly.

#### 2.9.2 Adverse event reporting and harms

All observed adverse events are recorded, reviewed by the study coordination team, and forwarded to the relevant ethics committees.

#### 2.9.3 Frequency and plans for auditing trial conduct

Project updates will be delivered annually to the funding organization as stipulated.

#### 2.9.4 Plans for communicating important protocol amendments to relevant parties (e.g., trial participants, ethical committees)

All major protocol revisions will be communicated to the ethics committees overseeing the participating sites.

### 2.10 Trial status

Protocol version 1.0. Recruitment began in summer 2025 and is expected to be completed in summer 2026. Trial registration: The trial was registered on May 22, 2025, in the German Clinical Trials Registry (Deutsches Register Klinischer Studien; Trial-ID: DRKS00034671).

### 2.11 Dissemination plans

The findings of the trial will be published in peer-reviewed journals. Furthermore, the results will be shared with healthcare professionals at both national and international conferences and communicated to the general public through appropriate lay media outlets.

## 3 Discussion

This study aims to evaluate the effectiveness of two psychotherapeutic interventions as add-ons to psychosomatic rehabilitation compared to treatment-as-usual alone for individuals PCS. The assessment tools used are designed to determine whether participants experience therapeutic benefits. PCS often manifests as a severe and persistent condition accompanied by various functional impairments and a considerable psychosocial burden (43). Moving beyond the traditional, often reductionist view on PCS – which tends to emphasize a unidimensional understanding and treatment – this study advocates for multimodal therapeutic strategies that address both the psychological and psychosocial dimensions of the condition. This approach is particularly pertinent given the supporting evidence for the effectiveness of psychotherapeutic methods in managing medically unexplained physical symptoms (44). Moreover, research suggests that, in certain patient subgroups at least, symptom severity may be more significantly impacted by the psychosocial consequences of the pandemic than by the viral infection itself (45). Consequently, various strategies have been developed to improve diagnostic accuracy in this area (46). Furthermore, patient feedback indicates a high level of satisfaction with such interventions (e.g. 10).

### 3.1 Strengths and limitations

PCS confronts society with productivity losses and rising healthcare costs, while the healthcare system is confronted with an increasing number of chronically ill patients and a greater need for long-term treatment. For this reason, it is highly relevant to implement appropriate treatment concepts. As two different interventions are being developed and researched in this project, it is possible to determine the extent to which even minor interventions or habits – such as reflecting on expectations – can promote general health. Previous results indicate that relatively little importance has been attached to this aspect in quantitative studies.

Another significant strength of this study is the use of cognitive tests to objectively assess and document the neurocognitive complaints experienced by patients with PCS. Given the often-subjective nature of cognitive symptoms reported by patients with PCS, the use of validated neuropsychological instruments allows for a thorough and objective evaluation of cognitive function. This approach not only helps to capture the complexity and variability of cognitive complaints across individuals, but it also provides a clearer picture of how PCS may affect different cognitive domains, such as attention, memory, executive functions, and processing speed. Furthermore, the study is pioneering in its examination of the role of personality traits in PCS, an area that has been largely underexplored in previous research. While there is evidence of cognitive and somatic symptoms of PCS, there is a clear lack of research on how personality functions may influence the course and experience of the condition. This project, therefore, makes an innovative and much-needed contribution to this field of research by investigating how specific personality traits may influence patients’ response to PCS and their overall recovery. Understanding these personality traits is crucial, as it may lead to more personalized therapeutic approaches that are better tailored to individual needs. This approach is consistent with the growing trend in healthcare to move towards more individualized and holistic treatment strategies to ensure that patients receive care that is better suited to their individual cognitive and psychological profiles. The WAI is the primary outcome of the study and will be used to assess participants’ work ability, providing a central measure of the effectiveness of the interventions under investigation.

Another strength is the integrative psychotherapeutic approach, which was developed in an iterative process by an interdisciplinary team from the coordinating institution. The two interventions take into account well-researched and established theories from the field of psychosomatics (biopsychosocial model, vulnerability-stress model, predictive coding, persistent somatic symptoms). In addition, findings from placebo and nocebo effect research are given special consideration. PoCo-FRAME is another study in the field of psychotherapy for patients with PCS and thus contributes to the further development of research that is still in its infancy.

Nonetheless, it is essential to discuss the extent to which this project is an efficacy or feasibility/pilot study. Given the advantages and disadvantages of the interventions, it would be desirable to follow up this work with an RCT. One limitation of the present study is that it primarily serves as a “proof of principle” rather than a fully validated intervention. While the study provides valuable initial insights into the effectiveness of the proposed approach, its scope and design may not yet allow for further generalizations or long-term conclusions. In particular, while the intervention implemented in this study is promising, it has not yet been tested in the context of more established, expectation-focused therapies. Therefore, the results of the current study should be interpreted as preliminary, and further research is needed to compare and integrate the intervention with existing, guideline-based treatments. Future studies could explore whether combining or adapting these approaches would improve patient outcomes and provide a more robust model for treating PCS.

Another limitation of the study is the non-standardized diagnosis of patients. Diagnoses are usually made by the patients’ attending physicians and registered for psychosomatic rehabilitation based on the diagnosis of PCS. The diagnoses are reviewed by rehabilitation physicians during the initial examination. For the study, the diagnoses are recorded from the discharge letter. However, for organizational and financial reasons, no standardized clinical interviews are conducted to confirm diagnoses. Furthermore, it would be desirable to conduct data collection at additional locations in order to increase the generalizability of the results; the implementation in only two rehabilitation clinics is a first step. With its 3-session approach, the manual offers a time-efficient treatment option, but it should of course to be viewed as an extremely small psychotherapeutic intervention. Given the severity of the impairment of some patients with PCS, it can be assumed, that – based on the clinical experience of those conducting the study –, more extensive interventions are necessary.

Although increasing importance is being placed on incorporating the patient perspective into research, it was unfortunately not possible to involve a patient advisory board (“lived experience”) due to time and organizational constraints. This would be highly desirable for future projects. Instead, patient feedback was gathered through other channels, such as consultations with experienced clinicians and informal input during the pilot phase. However, we are aware that having a dedicated advisory board would have further improved the development process. In future phases of the project, we plan to incorporate more structured patient feedback to optimize the relevance and acceptability of the intervention. We have trialed the intervention with test subjects and patients, and incorporated their feedback into our manual. Another limitation is that the information from the questionnaires is based on self-reports. The results may be distorted, especially when it comes to pension applications, for example. It would certainly also be informative to evaluate the interviews qualitatively or to review the journals. However, for ethical and methodological reasons, this is not possible in the current study and should be considered for follow-up projects. Another limitation of the study is that, due to organizational differences between clinics and funding, it is not yet possible to determine whether the interventions will be carried out by the same therapists who treat the patients or by therapists unknown to the patients. This potential inconsistency in the therapeutic relationship may affect the therapeutic alliance and the overall treatment experience, which could influence the results. To mitigate this issue, future studies could consider measures to ensure therapist continuity. We attempt to reduce this influence by training all study therapists in a standardized manner in the implementation of the manual.

Despite these limitations, the study provides important information about the far-reaching effects of PCS. For healthcare providers, this means taking patients seriously, creating interdisciplinary treatment options, and taking a differentiated approach to the syndrome. In addition, health policy strategies are needed to adequately address the long-term consequences of the pandemic, including rehabilitation and vocational reintegration. From a scientific perspective, further research on the etiology and therapeutic options is urgently needed.
